# Induced pluripotent stem cells carrying novel *APTX* mutations presented defective neural differentiation with the accumulation of DNA single-strand breaks

**DOI:** 10.1038/s41420-025-02723-2

**Published:** 2025-10-24

**Authors:** Zirui Chen, Yihua Huang, Zhirong Yuan, Kaibiao Xu, Yuqing Guan, Luqin Wang, Yawei Jiang, Weiling Deng, Yue Pan, Jing Liu, Yafang Hu

**Affiliations:** 1 Department of Neurology, Nanfang Hospital, Southern Medical University, Guangzhou, Guangdong province China; 2 Institute of Brain Disease, Nanfang Hospital, Southern Medical University, Guangzhou, Guangdong province China; 3 Center of Rehabilitation Medicine, Zhujiang Hospital, Southern Medical University, Guangzhou, Guangdong province China; 4 CAS Key Laboratory of Regenerative Biology, South China Institute for Stem Cell Biology and Regenerative Medicine, Guangzhou Institutes of Biomedicine and Health, Chinese Academy of Sciences, Guangzhou, Guangdong province China

**Keywords:** Experimental models of disease, Movement disorders, Disease genetics, Genetics research, Spinocerebellar ataxia

## Abstract

Ataxia with oculomotor apraxia type 1 (AOA1) is a rare, autosomal recessive, early-onset, progressive cerebellar ataxia caused by mutations in the *APTX* gene, which encodes aprataxin, a DNA-adenylate hydrolase involved in DNA damage repair. The pathogenesis of AOA1 remains unclear. The purpose of this study was to investigate the pathogenesis of a novel mutation, p.H201P/H201R, carried by our AOA1 patient and the mechanism of AOA1 in an induced pluripotent stem cells (iPSCs) model. We edited iPSCs derived from a healthy individual to carry the *APTX* homozygous mutation p.H201P (H201P-iPSCs) or p.H201R (H201R-iPSCs) via CRISPR/Cas9. We found that aprataxin expression was absent in both H201P- and H201R-iPSCs. The capacity of these *APTX*-mutant iPSCs to differentiate into neural progenitor cells (NPCs) and mature neurons was diminished. We observed an increase in DNA single-strand breaks (SSB) via a comet assay and poly(ADP-ribose) staining, and an increase in the ratio of cleaved PARP-1/total PARP-1 in *APTX*-mutant NPCs and early immature neurons (EiNs), in addition of a heightened sensitivity to tert-butyl hydroperoxide in *APTX*-mutant EiNs. Moreover, a decrease of APE1 expression was observed in *APTX*-mutant NPCs and H201R-EiNs during neural differentiation. Our study established a practical iPSCs model to investigate AOA1 disease. We found that mutant aprataxin leads to defective neural differentiation, accompanied by the accumulation of DNA SSBs with increased cleaved PARP-1 and reduced APE1 expression of the base excision repair pathway.

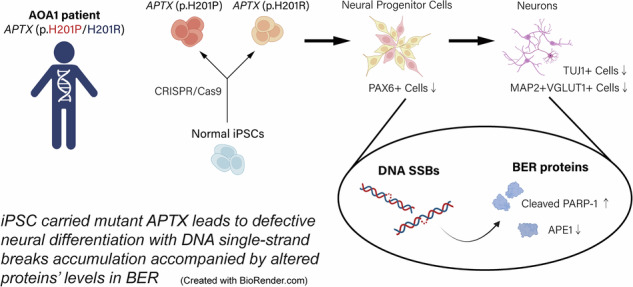

## Introduction

Ataxia with oculomotor apraxia type 1 (AOA1, MIM: 208920) is an autosomal recessive neurodegenerative disorder characterized by significant clinical heterogeneity. Clinical manifestations may include early-onset progressive ataxia, oculomotor apraxia, involuntary movement, peripheral neuropathy, and intellectual or psycho-behavioral abnormalities, along with hypoalbuminemia and hypercholesterolemia. The causative gene of AOA1 is *APTX*, which is located on chromosome 9 and encodes the protein aprataxin (APTX) [1]. APTX is located predominantly in the nucleus, with a minor presence in the cytoplasm and mitochondria [2, 3]. As a member of the DNA-adenylate hydrolase superfamily, APTX contains three important domains: an N-terminal forkhead-associated (FHA) domain for binding to X-ray cross-complementing protein 1 (XRCC1), XRCC4, and poly(ADP‒ribose) polymerase 1 (PARP-1) [4, 5]; a central histidine triad (HIT) domain with catalytic activity [6]; and a C-terminal putative zinc finger DNA-binding domain [7]. More than 40 mutations have been identified, of which most are nonsense or frameshift mutations, and missense mutations are usually located within HIT motifs [8]. These mutations often lead to APTX destabilization and a reduction in APTX expression, resulting in loss of function of aprataxin [9]. Substantial evidence points to distinct roles of APTX in base-excision repair (BER), which belongs to DNA single-strand break repair (SSBR) [10–12], and other studies have shown that APTX is involved in double-strand break repair (DSBR) [13]. SSBR impairment has been proven in APTX-knockdown cells [14], AOA1 patient-derived fibroblast lines and lymphoblast cell lines [11, 15]. These findings underscore the protective role of APTX in the DNA damage response. Additionally, coenzyme Q10 (CoQ10) deficiency in AOA1 patients with the p.W279X mutation is linked not to nuclear or mitochondrial DNA repair but rather to dysfunction of CoQ10 biosynthesis [16, 17]. Other studies suggest that APTX dysfunction contributes to mitochondrial dysfunction [18, 19] and abnormal immunological function [20, 21]. Thus, the pathogenesis of AOA1 remains incompletely understood.

Unfortunately, *Aptx* knockout mice do not recapitulate the progressive ataxia phenotypes and cerebellar atrophy characteristic of AOA1. However, when cellular antioxidant homeostasis is modulated by the expression of a mutant form of superoxide dismutase 1 (*SOD1*^G93A^) in an *Aptx*^−/−^ background, the number of spinal motor neurons decreases in *Aptx*^−/−^/*Sod1*^G93A^ mice [22]. Notably, *Aptx* and A-T-mutated (*Atm*) double knockout mice presented defects in cerebellar Purkinje cells, including cerebellar dysfunction, atrophy, and the development of progressive ataxia. These findings suggest that genotoxic stress contributes to neuronal death [23].

Interestingly, human induced pluripotent stem cells (iPSCs) have gained increasing recognition as disease models for in vitro experiments because of their ability to differentiate into diverse multipotent stem cells and mature somatic cells, such as neural progenitor cells (NPCs) and functional neurons [24, 25], while retaining the genetic characteristics of the provider [26]. Although an AOA1 patient-derived iPSC line derived from an AOA1 patient’s skin dermal fibroblasts has been generated [27], no further pathological phenotype has been analyzed.

In this study, we diagnosed a childhood-onset AOA1 patient carrying a novel compound heterozygous mutation in the *APTX* gene (c.602A>C/G, p.H201P/R). Accordingly, iPSCs carrying the *APTX* homozygous mutations p.H201P and p.H201R were generated through CRISPR/Cas9 gene editing of normal iPSCs. Our data demonstrate that the missense mutation of *APTX*, p.H201P or p.H201R, significantly impacts neuronal differentiation and DNA damage response. The *APTX*-mutant iPSCs constitute an alternative model for studying the mechanism of AOA1 disease.

## Results

### Clinical information of an AOA1 patient carrying a novel compound heterozygous mutation in *APTX*

Our patient, an 8-year-old boy of Chinese Han descent, presented with progressive unsteady walking and motor incoordination. He is the first child of a healthy couple with no known family history of gait disorders. The patient was born with normal development and started walking at the age of 1 year. However, he began to exhibit a drunk-like unsteady gait without convulsions at the age of 2 and a half. At age 3, magnetic resonance imaging of the cranium and cervical spine revealed no apparent abnormalities. Further diagnostic tests, including electroencephalography (EEG) and electromyography (EMG), also revealed normal results. The patient was initially diagnosed with dystonia by a pediatrician and was effectively managed with trihexyphenidyl hydrochloride tablets, although the effectiveness of the medication gradually decreased. Genetic analysis involving Sanger sequencing of the *TH* gene, associated with DOPA-responsive dystonia, and the *TOR1A* gene, linked to torsional dystonia type 1, yielded negative results. By age 6, his symptoms had worsened, characterized by inflexible hands and a tendency to fall while walking.

At the age of 8 years, he was referred to our outpatient center of the Department of Neurology. The neurological examination revealed unclear speech, communication reluctance, abnormal eye movement, increased muscle tone in the limbs, uncoordinated and imprecise movements, and a waddling gait. He was clinically diagnosed with extrapyramidal damage. The whole-exome sequencing analysis of the patient and his parents revealed that the patient carried a compound heterozygous mutation, c.602A>C (p. H201P, a novel missense mutation or variant with undetermined significance) and c.602A>G (p. H201R, a known pathogenic variant), in the *APTX* gene, each of which was inherited from his mother or father, respectively (Table 1). On the basis of the patient’s clinical presentation, genetic test results, and mode of inheritance of the disease, he was diagnosed with AOA1. Although CoQ10 treatment has been beneficial for some AOA1 patients, oral administration of CoQ10 did not improve our patient’s symptoms. Furthermore, the patient now requires assistance with a wheelchair. Compared with patients who carry known H201R [28] or H201Q [29] mutations, our patient presented an earlier age of onset and a more rapid progression of the disease (Table 2).

**Table 1:** Whole-exome sequencing reports of the patient and his parents.

Gene	Chromosome location	Transcript	Nucleotide alterations	Amino Acid alterations	Genotype	Carrier
*APTX*	chr9: 32984797	NM_175073.3	c.602A>C	p.H201P	Heterozygous	Father
			c.602A>G	p.H201R	Heterozygous	Mother
			c.602A>C/G	p.H201P/R	Compound heterozygous	Patient

**Table 2:** Comparison of the characteristics of patients who carried different mutations located at His201.

Mutation	H201P/H201R (Our patient)	H201R/H201R	H201Q/H201Q
Age of onset	2.5 years old	^a^7 years old, 8 years old	29 years old
Age at examination	8 years old	^a^48 years old, 44 years old	45 years old
Symptoms and Signs	Limb and truncal ataxia, waddling gait, communication reluctance and slurred speech, ocular motor apraxia, hypertonia	Limb and truncal ataxia, slurred speech, gaze nystagmus, external ophthalmoplegia, facial grimacing, choreiform movements, areflexia, distal muscle wasting, sensation disorders	Limb ataxia, imbalanced gait, dysarthria, nystagmus, muscle wasting, impaired position and vibration sense
Examination	Negative results in EEG, EMG, MRI (3 years old), without following examination	Hypoalbuminemia, hypercholesterolemia, reduced nerve conduction velocities (EMG), cerebellar atrophy (MRI), depletion of large myelinated fibers (Sural nerve biopsy)	Elevated serum creatine kinase, reduced nerve conduction velocities (EMG), cerebellar atrophy (MRI)
Treatment and effectiveness	Trihexyphenidyl hydrochloride, CoQ10; gradually decreased	Not mentioned	Not mentioned
Case source	This study	Shimazaki et al. [28]	Criscuolo et al. [29]

^a^The research reported two patients who had same clinical characteristics.

### Establishment of iPSCs with the novel mutation p.H201P and known mutation p.H201R in *APTX*, which results in defective aprataxin expression

**Fig. 1:**
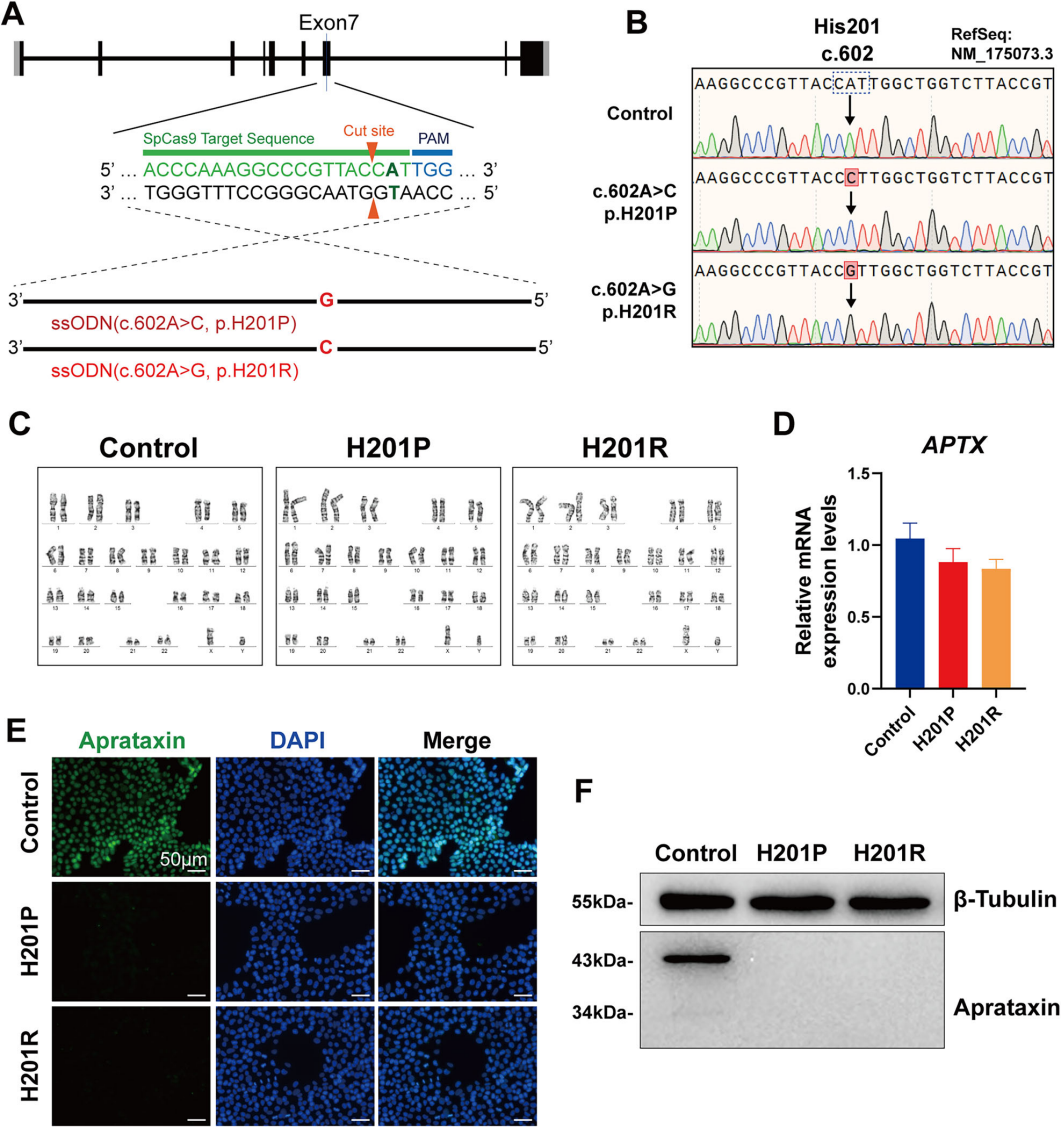
Generation and characterization of human iPSCs carrying *APTX* homozygous mutations via CRISPR/Cas9. **A** Schematic of the CRISPR-Cas9 strategy for genomic editing to generate iPSCs carrying *APTX* homozygous mutations (p.H201P and p.H201R). PAM: protospacer adjacent motif. **B** Representative Sanger sequences of *APTX* genes. Normal sequences and homozygous mutations edited by CRISPR/Cas9 are indicated by arrow tips and red highlights. **C** Normal karyotypes were observed for the control, H201P- and H201R-iPSCs. **D** qRT‒PCR analysis of the mRNA expression of *APTX* in iPSCs. The experiments were repeated three times (*n* = 3, mean ± SD). One-way ANOVA. There were no significant differences. **E** Immunofluorescence staining for aprataxin in iPSC colonies. The cell nuclei were counterstained with DAPI. Scale bar: 50 μm. **F** Western blot analysis of aprataxin in iPSCs. The experiments were repeated three times (*n* = 3).

To evaluate the pathogenicity of *APTX* (p.H201P), H201P-iPSCs were genetically constructed via ssODN-mediated CRISPR/Cas9 technology [30]. Similarly, H201R-iPSCs carrying a known pathogenic variant were constructed as a positive control (Fig. 1A). The related *APTX* mutations in different groups of iPSCs were confirmed through Sanger DNA sequencing (Fig. 1B). iPSCs were successfully cultured in an undifferentiated state and maintained a normal karyotype (Fig. 1C). Next, we tested the expression of APTX in iPSCs. Although the mRNA expression levels of *APTX* were not significantly different across all types of iPSCs, as shown in Fig. 1D, APTX protein expression was not detected in *APTX*-mutant iPSCs (Fig. 1E, F). Taken together, these results demonstrate that we successfully established iPSCs carrying the *APTX* p.H201P and p.H201R mutations, both of which leads to the absence of APTX expression.

### *APTX*-mutant iPSCs maintain pluripotency

**Fig. 2:**
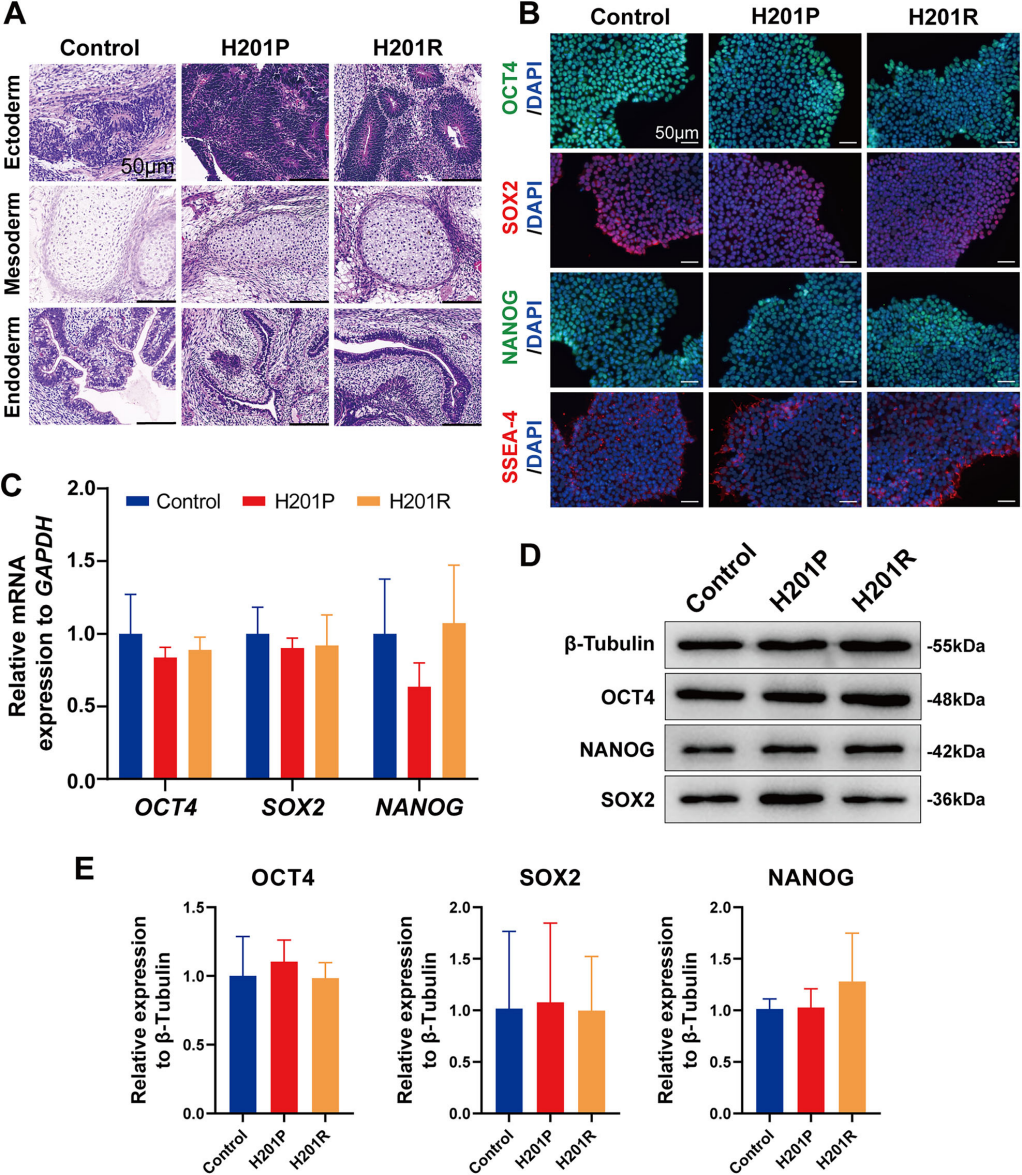
Human iPSCs carrying *APTX* homozygous mutations are pluripotent. **A** Teratoma formation in NOD-SCID mice following iPSCs injection. Tissue types from three germ layers—the ectoderm, mesoderm and endoderm—were observed. Scale bar: 50 μm. **B** Immunofluorescence staining for pluripotency markers of the iPSCs colonies: OCT4, SOX2, NANOG, and SSEA-4. The cell nuclei were counterstained with DAPI. Scale bars: 50 μm. **C** qRT‒PCR analysis of the pluripotency marker genes *OCT4, SOX2*, and *NANOG* in iPSCs. The experiments were repeated three times (*n* = 3, mean ± SD). One-way ANOVA. There were no significant differences. **D** Western blot analysis for pluripotency marker proteins of iPSCs: OCT4, SOX2, and NANOG. **E** The quantitative data from the Western blot for pluripotency marker proteins of iPSCs are presented in Fig. 2D. The experiments were repeated three times (*n* = 3, mean ± SD). One-way ANOVA. There were no significant differences.

We subsequently confirmed the ability of *APTX*-mutant iPSCs to spontaneously differentiate into cell types representing all three germ layers in vitro (Fig. 2A). Additionally, we verified the expression of the pluripotency markers OCT4, SOX2, NANOG, and the glycolipid carbohydrate epitope SSEA-4 via immunofluorescence staining (Fig. 2B). qRT‒PCR at the mRNA level (Fig. 2C) and Western blotting at the protein level (Fig. 2D, E) revealed no significant differences in OCT4, SOX2, or NANOG expression among the three groups. Taken together, these results demonstrate that *APTX*-mutant iPSCs maintain pluripotency and possess similar differentiation potential to that of control-iPSCs.

### *APTX*-mutant iPSCs are deficient in neural differentiation

**Fig. 3:**
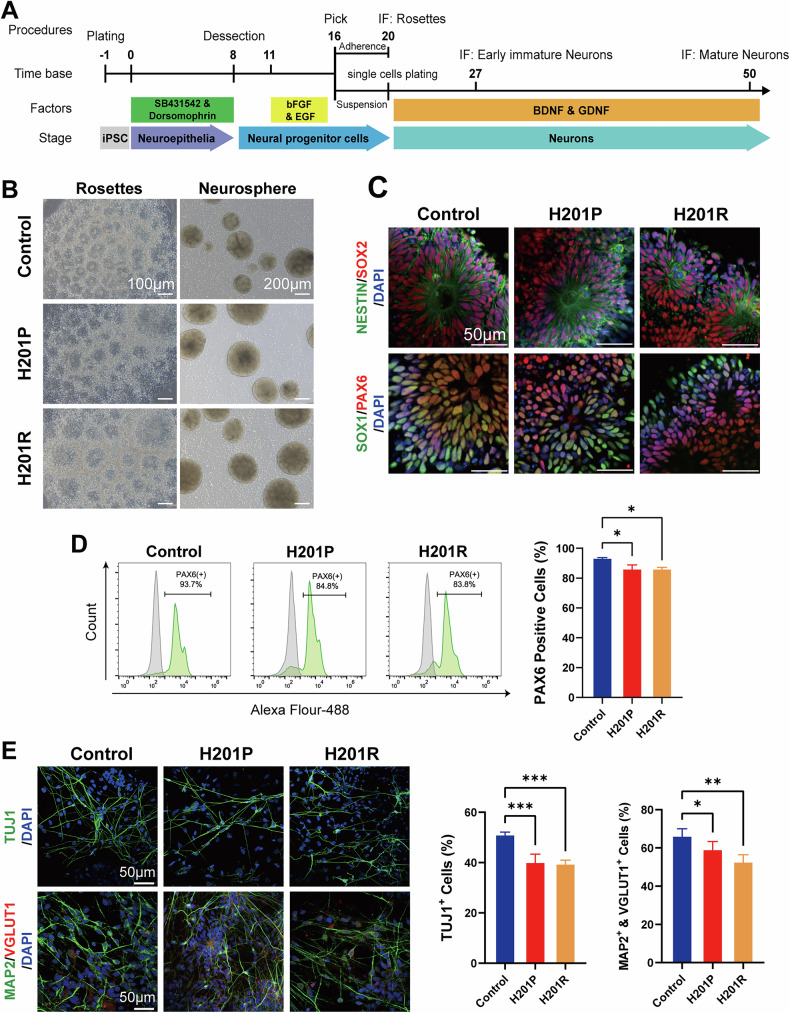
Human iPSCs carrying *APTX* homozygous mutations are defective in neural differentiation. **A** Schematic of the neural differentiation strategy for iPSCs (more detailed information is provided in the “Methods” section). **B** Morphology of the neural rosettes and neurospheres derived from iPSCs during neural differentiation on day 20. Scale bars in the left panel: 100 μm; scale bars in the right panel: 200 μm. **C** Immunofluorescence staining for the NPC markers NESTIN, SOX2, SOX1 and PAX6 in neural rosettes on day 20. The cell nuclei were counterstained with DAPI. Scale bar: 50 μm. **D** Flow cytometry analysis of PAX6-positive cells on day 16 during neural differentiation in the indicated groups. The experiments were repeated three times (*n* = 3, mean ± SD). One-way ANOVA. *P < 0.05. **E** Immunofluorescence staining for markers of early immature neurons, TUJ1, on day 27 and for markers of glutamatergic neurons, MAP2 and VGLUT1, on day 50 during neural differentiation (left). Nuclei were counterstained with DAPI. Scale bar: 50 μm. Quantitative data of TUJ1-positive neurons on day 27 and MAP2 and VGLUT1 double-positive cells on day 50 during neural differentiation were analyzed (right). The experiments were repeated four times (*n* = 4, mean ± SD). One-way ANOVA. *P < 0.05; **P < 0.01; ***P < 0.001.

To develop neuronal models, we induced iPSCs into NPCs via a modified dual SMAD inhibitor protocol, as illustrated in Fig. 3A [31, 32]. During the NPC stage of differentiation, *APTX*-mutant iPSCs were able to exit the pluripotency stage, displayed typical formation of neural rosettes and neurospheres (Fig. 3B), and expressed neural fate-related markers, including NESTIN, SOX2, SOX1 and PAX6 (Fig. 3C). As shown in Fig. 3D, flow cytometry analysis of NPCs on day 16 revealed a significant mild decrease in PAX6 in *APTX*-mutant iPSCs. We further induced the neuronal differentiation of these NPCs for 1 month. As expected, control NPCs successfully developed into early immature neurons (EiNs) and mature neurons. However, as shown in Fig. 3E, *APTX*-mutant NPCs showed impaired neuronal differentiation, as evidenced by immunostaining of specific markers, with a noticeable reduction in the ratio of β-III tubulin (TUJ1)-positive early immature neurons, and microtubule-associated protein 2 (MAP2) and vesicular glutamate transporter 1 (VGLUT1) co-positively expressed glutamatergic neurons. Taken together, these results demonstrate that *APTX*-mutant iPSCs are deficient in neural differentiation, including the NPC stage and the neuronal maturation stage.

### Absence of APTX leads to increased DNA single-strand breaks during neural differentiation

**Fig. 4:**
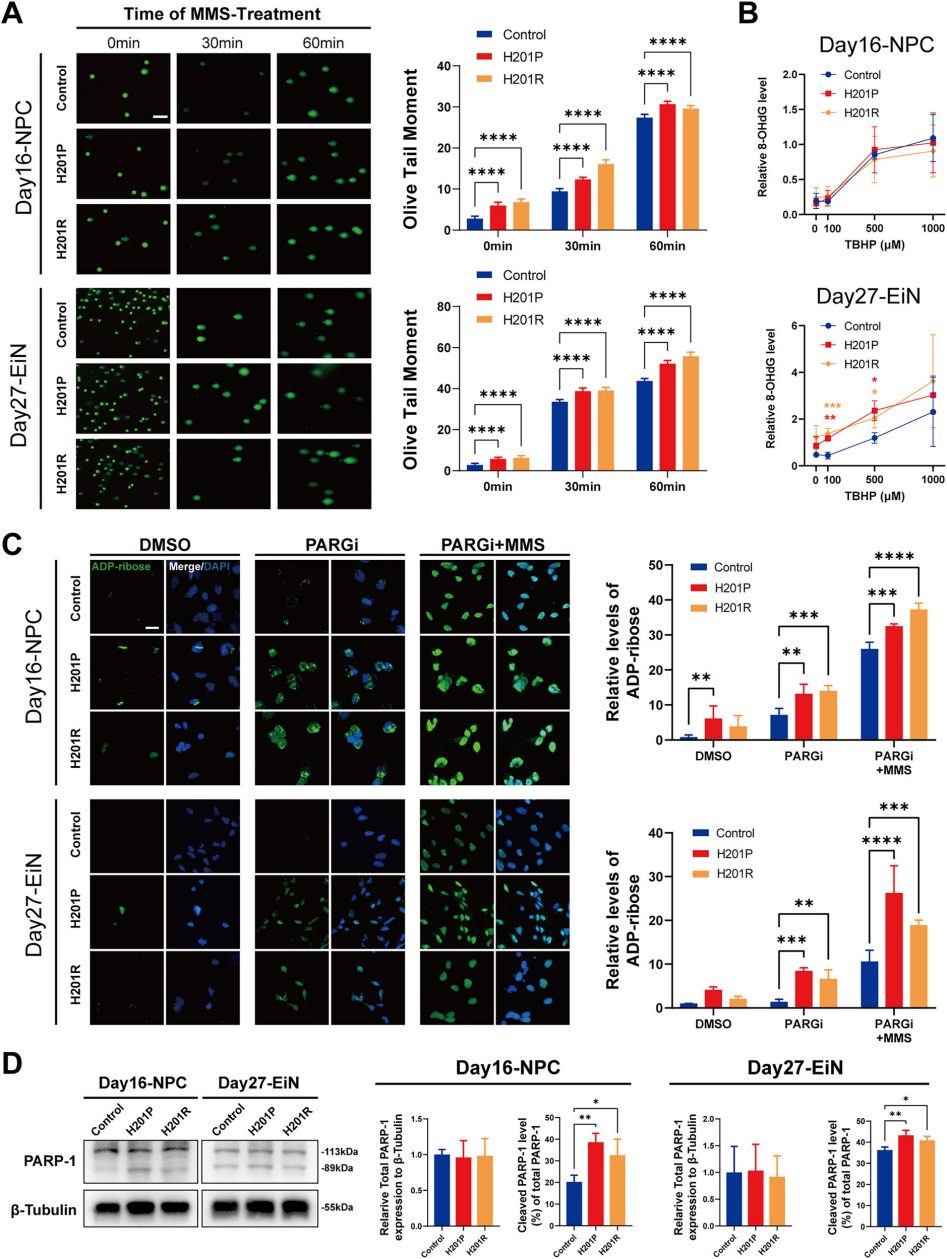
Increased DNA SSBs in iPSCs carrying *APTX* homozygous mutations during neural differentiation. **A** Comet assay images for NPCs on day 16 and EiNs on day 27 after 0, 30, and 60 min incubation with MMS during neural differentiation (left). Scale bar, 200 μm. The quantitative data for each group with different times of MMS treatment were analyzed (right). The experiments were repeated four times (*n* = 4, mean ± SD). Two-way ANOVA. ****P < 0.0001. **B** ELISA analysis of 8-OHdG in NPCs on day 16 and in EiNs on day 27 after 4 hours treatment with 0, 100, 500 and 1000 μM TBHP during neural differentiation. The experiments were repeated three times (*n* = 3, mean ± SD). Two-way ANOVA. *P < 0.05; **P < 0.01; ***P < 0.001. **C** Immunofluorescence staining for ADP-ribose of NPCs on day 16 and EiNs on day 27 after 30 min incubation with DMSO vehicle or PARG inhibitor (PARGi), or PARGi with MMS during neural differentiation (left). Scale bar, 20 μm. The quantitative data for each group were analyzed (right). The experiments were repeated four times (*n* = 4, mean ± SD). Two-way ANOVA. **P < 0.01; ***P < 0.001; ****P < 0.0001. **D** Western blot image of PARP-1 in NPCs on day 16 and in EiNs on day 27 during neural differentiation (left). The quantitative data of PARP-1 was analyzed (right). The experiments were repeated three times (*n* = 3, mean ± SD). One-way ANOVA. *P < 0.05; **P < 0.01.

Owing to the role of APTX in the DNA repair mechanism, we investigated whether neural differentiation from *APTX*-mutant iPSCs is associated with the accumulation of DNA damage. The comet assay was conducted on cells undergoing neural differentiation on days 16 and 27, which correspond to significant stages of NPCs and EiNs, respectively (Fig. 4A). The Olive tail moment (OTM) serves as indicators of DNA damage. Both *APTX*-mutant NPCs and EiNs presented elevated OTM values compared with those of the control group, indicating the accumulation of DNA damage. Additionally, we incubated the cells with alkylating agent methyl methanesulfonate (MMS) to increase DNA damage. Consistently, both *APTX*-mutant NPCs and EiNs presented elevated OTM values compared with those of the control group, indicating increased DNA breaks and compromised DNA repair capacity. We also examined 8-hydroxydeoxyguanosine (8-OHdG) [33] concentrations to identify sensitivity to oxidative DNA damage in the NPCs and EiNs treated with 0, 100, 500 or 1000 μM of tert-butyl hydroperoxide solution (TBHP), a strong oxidizer, for 4 hours [34]. In the stage of NPCs, there were no significant differences found among the three groups. However, significant elevated 8-OHdG levels were detected in *APTX*-mutant EiNs with 100 μM and 500 μM TBHP treatment (Fig. 4B), indicating higher sensitivity of oxidative DNA damage during early neuronal differentiation and maturation. Next, we assessed DNA double-strand breaks (DSB) in cells induced by long-time (90 min) treatments of MMS indicating by phosphorylated Ser-139 H2A.X (γH2AX) [35]. No difference was found in MMS-treated or untreated cells, indicating that *APTX*-mutant iPSCs do not generate endogenous DSB or alter the sensitivity of DSB during neural differentiation (Fig. S1A).

Literature report suggests that APTX involves in DNA SSBR, especially BER, as the primary modality [36]. To evaluate whether endogenous DNA single-strand breaks (SSB) impact cells undergoing neural differentiation, we attempted to detect endogenous poly(ADP-ribose) (PAR) in cells following 30 min incubation with a poly(ADP-ribose) glycohydrolase inhibitor (PARGi) [37]. Both *APTX*-mutant NPCs and EiNs exhibited elevated PAR levels compared with control groups when PAR degradation was prevented by PARGi. Strikingly, NPCs of H201P groups presented significantly higher PAR level than in the control group treated with DMSO vehicle (Fig. 4C). These data suggested that there was a subset of cells with high SSBs. Moreover, we triggered DNA SSBs by short-time (30 min) MMS based on PARGi treatment. Expectedly, both *APTX*-mutant NPCs and EiNs lighted up with higher PAR compared with control groups (Fig. 4C), indicating higher levels of PARP activity and more unrepaired SSBs. PARP-1 and AP endonuclease 1 (APE1) are upstream components of APTX, while XRCC1 and DNA polymerase beta (Polβ) are downstream components of the short-patch pathway; flap endonuclease-1 (FEN1) is involved in the long-patch pathway of BER [38]. Furthermore, we examined the expression levels of DNA repair proteins involved in BER above in these cells on days 16 and 27 via Western blot analysis. In the stage of NPCs, there were no statistically significant differences in the total expression of PARP-1, XRCC1, DNA Polβ, or FEN1 among the three groups (Figs. 4D and S1B). However, the ratio of cleaved PARP-1 increased (Fig. 4D), whereas the level of APE1 expression decreased at this stage (Fig. S1B). Similarly, in the stage of EiNs, the ratio of cleaved PARP-1 increased (Fig. 4D) whereas only the H201R group displayed a reduced level of APE1 expression (Figure S1B). Taken together, these data demonstrate that *APTX*-mutant iPSC-derived NPCs and EiNs exhibit increased DNA SSBs with altered level of cleaved PARP-1 and APE1, which are involved in the BER pathway during neural differentiation.

## Discussion

AOA1 is a rare disorder characterized by unclear pathogenic mechanisms and a lack of suitable disease models; it falls under the category of hereditary ataxias associated with impaired DNA repair function and presents with a rare onset [39]. In this study, we present a case of AOA1 in a patient with a compound heterozygous mutation (p.H201P/H201R) in the HIT motif of the *APTX* gene, where p.H201P is a novel missense mutation. The patient exhibited rapidly progressive ataxia with childhood onset, severe impairment in motor coordination, and oculomotor apraxia. To investigate the mechanisms of AOA1 and the pathogenicity of the p.H201P/R mutations, we generated two iPSCs lines carrying *APTX* homozygous H201P or H201R mutations, all of which lacked APTX expression. We found that H201P-iPSCs and H201R-iPSCs displayed moderate degrees of impairment in neural differentiation at the NPCs, EiNs, and mature neurons stages, accompanied by the accumulation of DNA SSBs and altered levels of cleaved PARP-1 and APE1, which are involved in the BER pathway. Therefore, we successfully generated an *APTX*-mutant iPSC disease model for studying the cellular and molecular mechanisms of neuronal differentiation, which may serve as an alternative model for testing potential treatments.

Here, we performed a comparison study with two *APTX-*mutant iPSCs carrying homozygous H201P or H201R. Missense mutations are usually located on the HIT motif [8]. Crystal structure analysis revealed that the 201 histidine amino acid residue plays a critical role in both the catalytic activity and structural stability of the protein, whereas experimental studies verified the loss of solubility of APTX for both the H201R and H201Q mutations, suggesting that the H201R mutant is highly unstable and that H201Q has moderate instability [9], which is accompanied by early onset and late onset, respectively. In agreement with these observations, both *APTX-*mutant iPSCs lacked APTX protein, indicating that the pathogenic mutation H201P has high instability. Since we found that the two *APTX-*mutant iPSCs had *APTX* mRNA levels similar to those of the control, these mutations might primarily disrupt posttranscriptional processes or increase protein instability. When the severity of phenotype of *APTX-*mutant iPSCs through differentiation (Fig. 3) and DNA damage (Fig. 4) was compared, H201R-iPSCs presented similar impairments as H201P-iPSCs.

Importantly, our study provides dynamic observations of the effects of mutant APTX on neuronal differentiation and the underlying molecular mechanisms involved. First, our study revealed that *APTX-*mutant iPSCs had mild differentiation deficits early in the NPCs stage, representing the differentiation of the neuroepithelium into the neural tube [40, 41]. This observation might indicate potentially developmental disorder contributes to early onset of the disease. Second, during subsequent differentiation into early immature neurons, both *APTX-*mutant cell types diminished in terms of the ability to form immature neurons. As NPCs differentiate into neurons and their neuronal function matures, they display a slower rate of self-renewal and proliferation [41]. This process requires increased DNA repair and transcriptional regulatory activities compared with those in other tissues [42, 43]. Our comet assay revealed increased DNA damage in *APTX-*mutant NPCs and EiNs. Consequently, a disturbance in DNA repair mechanisms may lead to compromised neuronal maturation and viability. Wang et al. demonstrated that the process of active demethylation in neurons results in greater generation of DNA SSBs than other cell types do [44]. Another significant factor contributing to DNA SSBs is oxidative stress [36]. Notably, we observed that *APTX*-mutant EiNs but not NPCs exhibited heightened sensitivity to TBHP. This stage-specific vulnerability likely arises from the metabolic switch that occurs during the differentiation of NPCs into neurons, shifting from glycolysis to mitochondrial oxidative phosphorylation [45], which results in an increased production of reactive oxygen species and elevated oxidative stress, consequently. The DNA SSBs are recognized by PARP-1 and recruited by aprataxin to participate in the repair process [46, 47]. Defects in APTX may result in decreased efficiency of DNA SSB repair [12, 14, 48], suggesting the potential accumulation of SSBs and an increase in the PARP-1 activity. Importantly, we found *APTX-*mutant NPCs and EiNs exhibited higher level of ADP-ribose treated by PARGi under the treatment of MMS or not. Although we did not observe increased PARP-1 content during the neural differentiation of *APTX-*mutant iPSCs, interestingly, we detected a greater proportion of cleaved PARP-1/total PARP-1 in *APTX-*mutant neurons. PARP-1 acts as a substrate for cleaved caspase-3 and facilitates apoptosis following cleavage [49, 50], indicating increased apoptosis after interaction with the DNA SSB nick in this study. APE1 is an important enzyme that participates in the BER pathway of DNA SSBR. It cleaves abasic site (also known as apurinic/apyrimidinic site, AP site), leaving a 5ʹ-terminal sugar gap that is detected by PARP-1, resulting in an SSB intermediate [51]. APE1 then acts as a loading factor for DNA Polβ onto non-incised AP sites in DNA and stimulates the 5’-terminal deoxyribose 5’-phosphate excision activity of DNA Polβ [52], which plays a key role in base-excision repair and interacts with FEN1 and XRCC1 [53, 54]. Eventually, similar to previous studies [17, 55], we observed reduced APE1 expression in *APTX-*mutant NPCs as well as in H201R-EiNs, which may suggest that increased accumulation of DNA SSBs could be associated with altered efficiency of BER. In addition, we examined the levels of γH2AX, which were not significantly different from those of the controls, suggesting mere DNA DSBR are not impact in these *APTX-*mutant iPSCs during neural differentiation. These data agree with the findings in the animal carrying mutant *Atm* and *Aptx* genes, which revealed that broad DNA impairment is sufficient to lead to disease. Overall, the *APTX-*mutant iPSCs disease models we established allow us to elucidate the impact of loss of function of APTX on the biological process of neurons and the underlying mechanisms involved.

Our study has several limitations. First, we generated iPSCs that carried *APTX* homozygous mutations from only one AOA1 patient but no other known pathogenic variants. Second, a greater number of iPSC passages may increase the risk of mutations that impair differentiation capacity [56]. Third, we used a dual SMAD inhibitor regimen to generate cortical neurons but not Purkinje neurons, which would better mimic the pathological features of AOA1 patients. Therefore, differentiating iPSCs to generate cerebellar organoids might better mimic disease progression in patients [57, 58]. This strategy requires a longer differentiation period and produces a more complex neurological tissue component [59, 60]. Finally, we did not construct a patient-derived iPSCs model or explore *APTX* gene therapy as a potential treatment for AOA1. Future research should focus on comparisons of different mutations located in both His201 and others through neural differentiation into cerebellar organoids. In addition, analysis of a patient-derived iPSCs model could provide strong evidence of the pathological mechanism of AOA1.

In conclusion, we generated iPSCs harboring homozygous *APTX* mutations: H201P or H201R. Our findings indicate that these *APTX-*mutant iPSCs exhibit deficits in neural differentiation and that the accumulation of DNA SSBs accompanied by altered levels of cleaved PARP-1 and APE1 involved in the BER pathway, which may elucidate the molecular mechanisms of AOA1.

## Materials and methods

All experimental procedures were performed in accordance with the guidelines and regulations of Nanfang Hospital, Southern Medical University, and Guangzhou Institutes of Biomedicine and Health, Chinese Academy of Sciences.

### Clinical case

The study was approved by the Ethical Review Committee at Nanfang Hospital, Southern Medical University, Guangzhou, China, on September 28, 2023 (approval number: NFEC-2023-427). All participants provided informed consent. The history of symptoms and signs as well as the neurological examination and other clinical procedures were applied as standardized by Nanfang Hospital, Southern Medical University. Whole-exome sequencing was conducted and analyzed at Amcarelab Medical Testing Ltd., Guangzhou, China.

### Culture and maintenance of human iPSCs

Human iPSCs were reprogrammed from peripheral blood mononuclear cells gifted by Professor Pan Lab in accordance with the guidelines of the Human Subject Research Ethics Committee at Guangzhou Institutes of Biomedicine and Health (GIBH), Chinese Academy of Sciences (CAS). To maintain iPSCs, mTeSR Plus medium (STEMCELL Technologies, Vancouver, BC, Canada) and Matrigel (#354230, Corning, Glendale, AZ)-coated plates were used. The cells were passaged every 3 days, digested with Accutase (STEMCELL Technologies), replaced with fresh medium every day, and maintained at 5% CO_2_ and 37 °C.

### Gene editing via CRISPR/Cas9 in human iPSCs

The pX330-2A-Puro plasmid (GIBH-CAS, Guangzhou, China), which contains puromycin resistance gene cassettes, can express the Cas9 protein and single guide RNA (sgRNA). The sgRNA sequences targeting *APTX* were designed on the CRISPOR website (http://crispor.tefor.net/), with the cut site located 1 base pair away from the mutant base: Forward: 5’-CACCGACCCAAAGGCCCGTTACCAT-3’; Reverse: 5’-AAACATGGTAACGGGCCTTTGGGTC-3’. Single-stranded oligodeoxynucleotides (ssODNs) containing specific mutant bases were designed to generate *APTX* homozygous mutations c.602A>C (p. H201P) [5’-CACAGTGTGCATATGCTTAAGGAGTTCAAGGTGTTCCCTGGCCACAGCCTTCAGACTGGAAATGGAGGTCCACGGTAAGACCAGCCAAGGGTAACGGGCCTTTGGGTATTTATCCTTTATCACCAC-3’] and c.602A>G (p. H201R) [5’-CACAGTGTGCATATGCTTAAGGAGTTCAAGGTGTTCCCTGGCCACAGCCTTCAGACTGGAAATGGAGGTCCACGGTAAGACCAGCCAACGGTAACGGGCCTTTGGGTATTTATCCTTTATCACCAC-3’], respectively. For targeting, 1×10^6^ iPSCs were electroporated with 500 pmol of ssODNs and 4 μg of pX330 plasmid containing sgRNA for each gene via a Human Stem Cell Nucleofector Kit (#VPH-5022, Lonza, Basel, Switzerland). The electroporated iPSCs were subsequently plated onto Matrigel-coated six-well plates supplemented with 10 μM Y-27632 (Chemleader, Shanghai, China) for 1 day. Positively expressed cells were selected with 1 μg/mL puromycin (Gibco, Grand Island, NY) in mTeSR Plus for 48 hours. The surviving cells were propagated into clones, collected, and analyzed via Sanger DNA sequencing (GENEWIZ, Suzhou, China).

### Teratoma formation

The teratoma formation experiments were approved by the Ethical Review Committee on animal experiments and performed according to the guidelines of Animal Care at the Guangzhou Institutes of Biomedicine and Health, Chinese Academy of Sciences, on July ^11^, 2021 (approval number: IACUC-2021030). The animal experiments were performed with approval and according to the guidelines of the Animal Care and Use Committee of the GIBH. All participants provided informed consent. Each type of iPSC was injected into two 4-week-old female NOD-SCID mice (Gempharmatech, Jiangsu, China) in biological replicates. A total of 1×10^6^ iPSCs mixed with Matrigel were subcutaneously injected into one side of the armpit and thigh of each mouse, and teratomas formed from 4--8 weeks. To euthanize the mice, CO_2_ was used in a special euthanasia box. Teratoma tissues were collected and then embedded in paraffin, sectioned, and subjected to hematoxylin-eosin staining in accordance with the guidelines of approved protocols of pathology lab at GIBH-CAS.

### Neural differentiation

Human iPSCs at 100% confluence were seeded onto Matrigel-coated 12-well plates in mTeSR Plus medium supplemented with 10 μM Y-27632. After 1 day (defined as day 0), the culture medium was changed to N2B27 medium: 50% DMEM/F12 (Thermo Fisher Scientific) with 1× N2 (Gibco), 50% neurobasal medium (Gibco) with 1× B27 (Gibco), 1× GlutaMAX, 1× NEAA and 5 μg/mL insulin (Sigma) plus 10 μM SB431542 (Selleck, Houston, TX) and 1 μM dorsomorphin (Selleck). The medium was changed every day. On day 9, the cells were passaged at a 1:2 ratio on new Matrigel-coated 12-well plates in N2B27 medium supplemented with 10 μM Y-27632. The medium was subsequently changed to fresh N2B27 medium every 2 days. From day 11 to day 15, the cells were cultured with N2B27 medium supplemented with 20 ng/mL bFGF. On day 16, rosette-like neural progenitor cell (NPC) colonies were picked. These clones were subsequently seeded into ultralow adhesion 6-well plates in N2B27 medium supplemented with 10 μM Y27632, which was subsequently changed to N2B27 medium the next day. On day 20, neurospheres containing canonical neural rosettes appeared. Then, the neurospheres were collected and digested into single cells with Accutase for neuronal differentiation. Neural progenitor cells (1 × 10^4^/cm^2^) were seeded onto poly-L-lysine (Sigma)/Laminin (Sigma)-coated plates in N2B27 medium supplemented with 10 μM Y-27632. On day 21, the culture medium was changed to N2B27 medium supplemented with 20 ng/mL BDNF (PeproTech) and 20 ng/mL GDNF (PeproTech). The medium was changed every 2 days. Further differentiation continued until day 50, when the canonical morphology of the neurons appeared. We conducted independently experimental repeats from the beginning of the differentiation program, and measured the different batch of differentiated neural cells.

### Quantitative real‑time PCR (qRT‑PCR)

Total RNA was extracted from the cells with TRI reagent (MRC, Cincinnati, OH). Briefly, 1 μg of total RNA was reverse transcribed with a HiScript III RT SuperMix for qPCR Kit (Vazyme, Nanjing, China). We subsequently performed a qRT‒PCR assay with ChamQ SYBR qPCR Master Mix (Vazyme) and Applied Biosystems QuantStudio 3 (Thermo Fisher Scientific). We used *GAPDH* to normalize the qRT‒PCR results for our samples. All the data were analyzed with three replicates. All primer sequences are listed in Supplementary Table 1.

### Immunofluorescence staining assay

Human iPSCs on day 3 were used for the immunofluorescence staining assay. On day 16, NPCs were transferred to Matrigel-coated confocal cover glass bottom dishes (35 mm, Biosharp, Hefei, China) and cultured for 4 days in N2B27 medium for immunofluorescence staining. Single NPCs on day 20 were seeded onto poly-L-lysine/Laminin-coated confocal cover glass bottom dishes and differentiated into neurons on days 27 and 50 for immunofluorescence staining. As for ADP-ribose staining, NPCs and neurons were incubated for 30 min with DMSO vehicle or 10 μM PARG inhibitor (PDD 0017273; AmBeed, Chicago, IL), or 10 μM PARG inhibitor plus 0.2 mg/mL MMS (Macklin, Shanghai, China). The cells were subsequently fixed in 4% paraformaldehyde (PFA; Beyotime, Beijing, China) for 30 min at room temperature. After being washed three times with PBS (Elgbio, Guangzhou, China) for 5 min each time, the cells were permeabilized and blocked with 0.25% Triton X-100 (Sigma) and 5% bovine serum albumin (BSA, Sigma) in PBS. Moreover, the cells were incubated with the corresponding primary antibodies overnight at 4 °C. After being washed three times with PBS for 5 min each time, the cells were incubated with the corresponding secondary antibodies for 1 h. After being washed four times with PBS for 5 min each time, DAPI (Sigma) was added to the incubation solution at room temperature for 5 min. After being washed two times with PBS for 5 min each time, images were captured with a Dragonfly 200 microscope (Andor, Belfast, UK) and an LSM 980 microscope (Zeiss, Oberkochen, Germany). For quantitative analysis, nuclei were identified based on the DAPI signal and were gated and quantified using *ImageJ* (NIH Image processing program). Numbers of neurons exhibiting positive cytoplasmic expression of TUJ1 or MAP2/VGLUT1 were gated by *ImageJ* using the positive fluorescence intensity of the control group served as a predefined fluorescence threshold criterion, and were manually counted based on nuclei surrounded by staining in both the neurites and cell bodies. At least 200 neurons from each group were analyzed per independent experiment. Fluorescence intensities of ADP-ribose were obtained by *ImageJ* measuring the cells that met the fluorescence threshold. The relative level of ADP-ribose was calculated as the total fluorescence intensity divided by the total number of nuclei, and the results were normalized against the control group treated with DMSO. At least 1000 nuclei for interphase cells from each group or condition were analyzed per independent experiment. The detailed information of the antibodies is listed in Supplementary Table 2.

### Western blot

The collected cells were lysed in RIPA buffer (Beyotime) containing protease and phosphatase inhibitor cocktail (Boster, Wuhan, China) on ice for 15 min. Twenty micrograms of total cellular protein were loaded onto 12% SDS‒PAGE gels. After electrophoresis, the samples on the SDS‒PAGE gels were transferred to PVDF membranes (Millipore, Billerica, MA), which were further incubated with primary antibodies overnight at 4 °C, washed three times with TBST, and then incubated with HRP-conjugated secondary antibodies at room temperature for 1 hour. The proteins in the final TBST-washed PVDF membranes were detected via enhanced chemiluminescence (ECL) (Bio-Rad, Hercules, CA) and visualized with a Chemi image analysis system (Bio-Rad). As for γH2AX detecting, cells were treated without or with 0.2 mg/mL MMS for 90 min. Detailed information on the antibodies is listed in Supplementary Table 2.

### Flow cytometry analysis

Cultured cells were digested into single cells with Accutase and then fixed in 4% PFA. After being washed with PBS, the cells were permeabilized in perm/wash buffer (0.1% Triton X-100 and 1% BSA in PBS) at 4 °C for 10 min. After being washed, the cells were divided into two equal parts: one was incubated with the corresponding primary antibodies at 37 °C for 30 min, and the other was incubated with the corresponding isotype control antibodies at 37 °C for 30 min. After being washed, the cells were incubated with secondary antibodies at 37 °C for 30 min, washed in PBS twice, resuspended in PBS, and analyzed with an Accuri C6 Plus flow cytometer (BD Biosciences, San Jose, CA). Ten thousand cells were gated and analyzed from each group. The detailed information on the antibodies is listed in Supplementary Table 2.

### Comet assay

An Oxiselect Comet Assay Kit (Cell-Biolabs, San Diego, CA) was used. Briefly, NPCs on day 16 and early immature neurons on day 27 were treated without or with 0.2 mg/mL MMS for 30 min or 60 min, respectively. All the cell samples were washed with cold PBS and gently removed from the plate well by scraping with a rubber policeman. The cells were transferred to conical tubes and centrifuged at 700×g for 2 minutes. The cell pellets were washed once with ice-cold PBS and centrifuged, and the cell pellets were resuspended in ice-cold PBS at 1 × 10^5^ cells/mL. The cell suspension was mixed with agarose at a 1:10 ratio (v/v). The mixture was placed on a slide and then incubated in the dark at 4 °C for 30 min. The slide was immersed in lysis buffer, incubated in the dark at 4 °C for 60 min, transferred to an alkaline solution, and incubated in the dark at 4 °C for 30 min. The slide was placed into an electrophoresis chamber and subjected to electrophoresis for 20 min in alkaline electrophoresis buffer at 300 mA and 1 volt/cm. After electrophoresis, the slide was washed 3 times with 70% ethanol for 5 min. The slide was incubated at 37 °C for 1 h. Vista Green DNA dye was added for 10 min, and the cells were observed and recorded via an inverted fluorescence microscope (Zeiss). *CASPlab* (Comet Assay Software Project) was used to analyze the “Tail moment” and “Tail DNA%” of the cells, and at least 50 cells were analyzed per condition from each group. Olive tail moment (OTM) is an appropriate index for measuring DNA damage. OTM = *Tail DNA%*×*Tail moment length*, Tail DNA% = 100%×*Tail DNA intensity*/*cell DNA intensity*. The tail moment length is measured from the center of the head to the center of the tail.

### Oxidative DNA damage test

Briefly, all groups of NPCs on day 16 and early immature neurons on day 27 were treated with indicated concentrations of TBHP (Macklin, Shanghai, China) for 4 h and then collected to extract genomic DNA via a HiPure Tissue DNA Mini Kit (Magen, Guangzhou, China). The levels of 8-OHdG of DNA samples were measured with an enzyme-linked immunosorbent assay (ELISA) kit (Elabscience Biotechnology, Wuhan, China) [61]. The optical density data were obtained via *Gen 5* system of BioTek Synergy H1 Multifunctional microplate detector (Agilent, Santa Clara, CA), and were converted to concentration values based on the standard curve from each experiment, divided by the sample DNA concentration, and the final results were normalized against the control group.

### Statistical analysis

The statistical data are presented as the means ± standard deviation (SD) or median with interquartile range and were calculated via Microsoft Excel and GraphPad Prism software (version 9.5) from at least three independently repeated experiments. One-way analysis of variance (ANOVA) followed by Dunnett’s *post hoc* analysis was used to determine the significance level between samples. Two-way ANOVA followed by Dunnett’s *post hoc* analysis was used to determine the significance level between grouped samples. *P* values < 0.05 were considered statistically significant. The results of the statistical tests were annotated in the figures with asterisks to indicate the level of significance.

## Supplementary information

Supplementary Information

Supplementary Figure S1

Supplementary Figure legends

Supplementary Table S1

Supplementary Table S2

Supplementary Uncropped Western Blot image

## Data Availability

The data that support the findings of this study are available from the corresponding author upon reasonable request.
