# Supplementary material for: Induced pluripotent stem cells carrying novel APTX mutations presented defective neural differentiation with the accumulation of DNA single-strand breaks
Source: Cell Death Discov. 2025 Oct 24;11:481. doi: 10.1038/s41420-025-02723-2 (PMC12552585; doi:10.1038/s41420-025-02723-2)
Supplement: Supplementary file 1 — Supplementary Information [file 41420_2025_2723_MOESM1_ESM.docx]

**Supplementary Information:**

**Supplementary Figure legends**, format: Word

This file includes legends of supplementary figure S1.

**Supplementary Figure S1**, format: Tiff

This file includes figures directly relevant to the result that cannot be included in the printed version owing to space constraint.

**Supplementary Table S1**, format: Excel

This file includes the sequences of the primers used for the sanger DNA sequencing and qPCR for this manuscript.

**Supplementary Table S2**, format: Excel

This file includes detailed information on the antibodies used for immunofluorescence staining and Western blotting in this manuscript.

**Supplementary Uncropped Western Blot image,** format: PDF

This file includes uncropped original Western blots used in this manuscript.
