# Supplementary figures and images for: Induced pluripotent stem cells carrying novel APTX mutations presented defective neural differentiation with the accumulation of DNA single-strand breaks
Source: Cell Death Discov. 2025 Oct 24;11:481. doi: 10.1038/s41420-025-02723-2 (PMC12552585; doi:10.1038/s41420-025-02723-2)

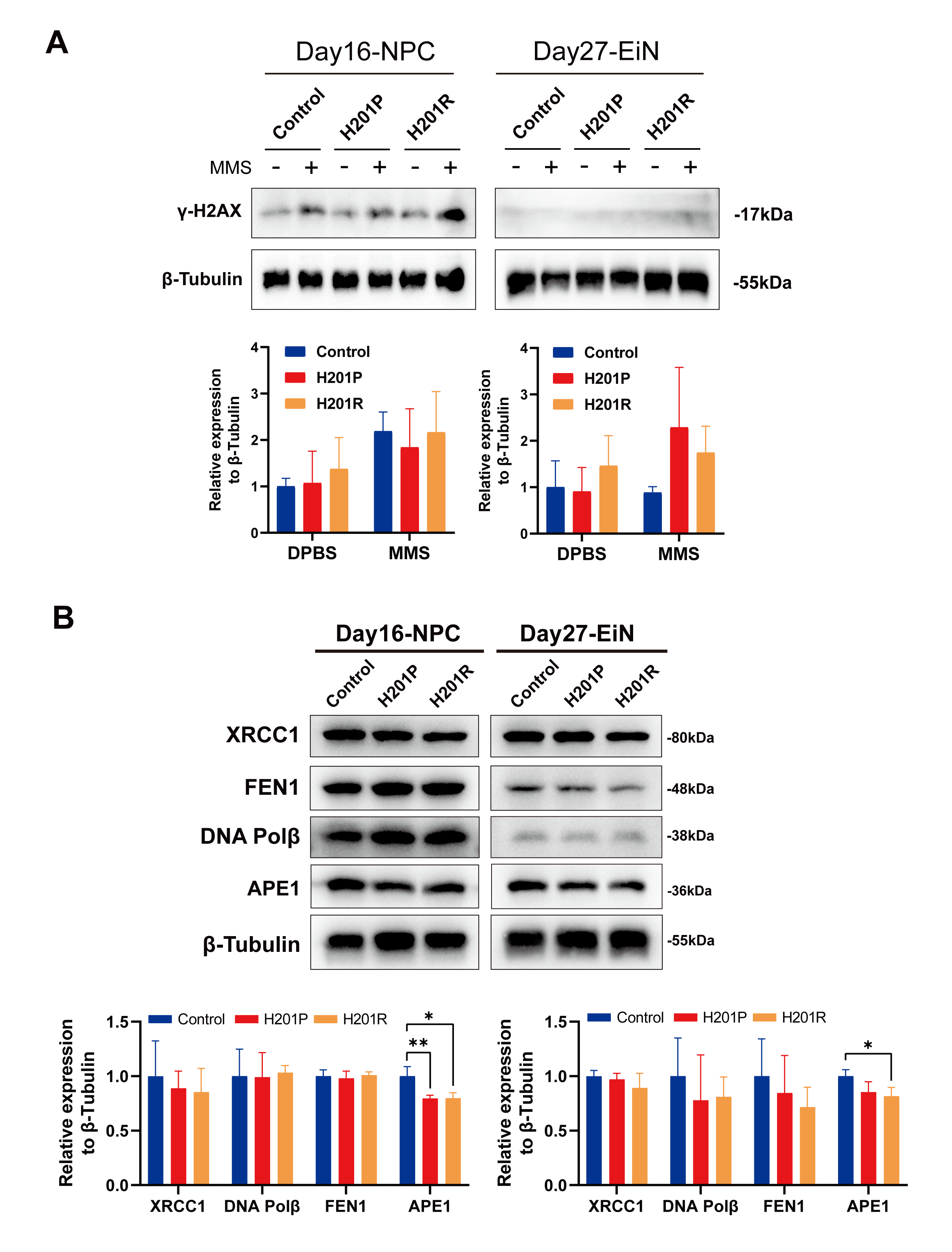

Supplement: Supplementary file 2 — Supplementary Figure S1 [file 41420_2025_2723_MOESM2_ESM.tif]

**Figure 1F**

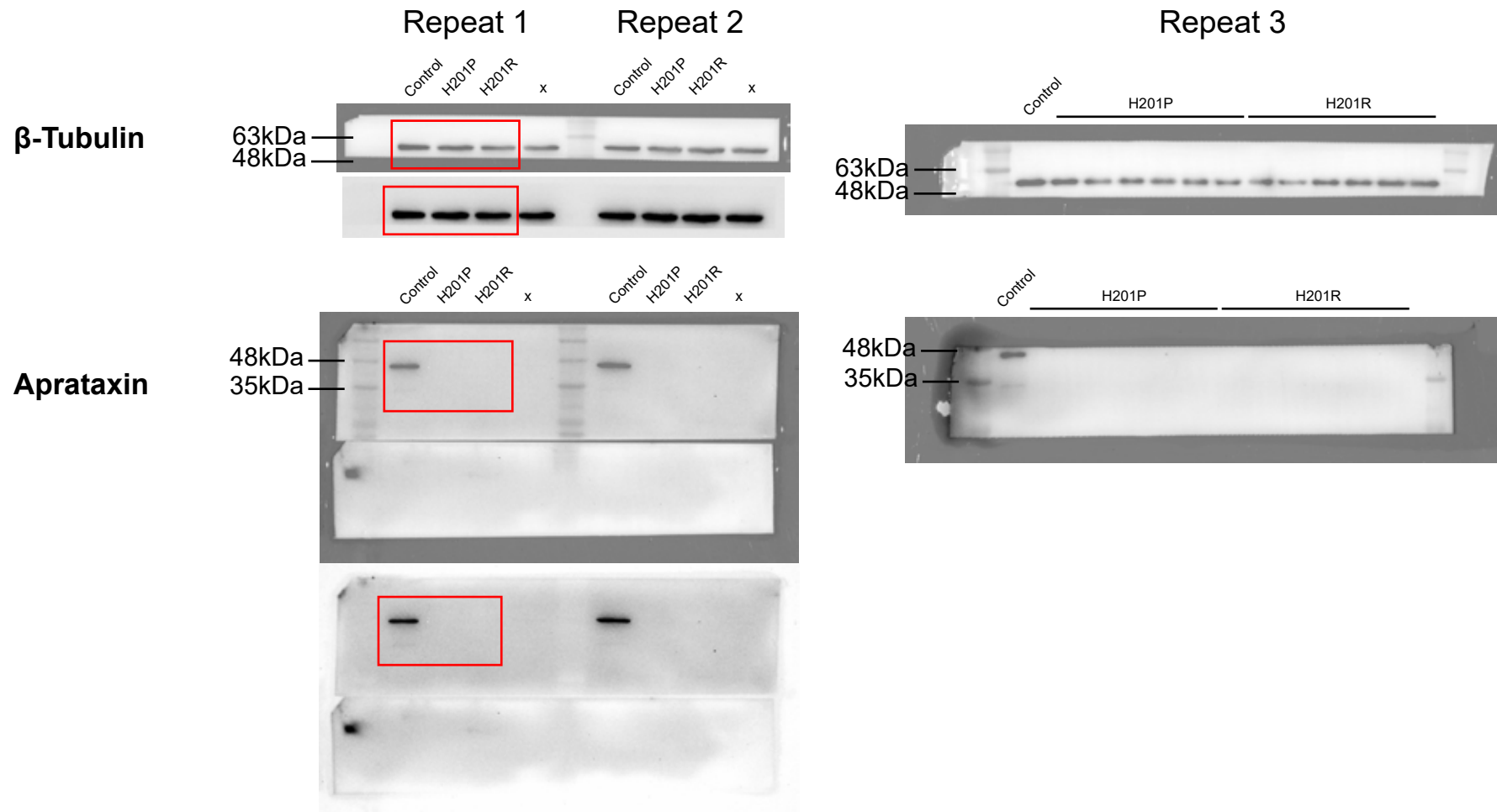

**Figure 2D**

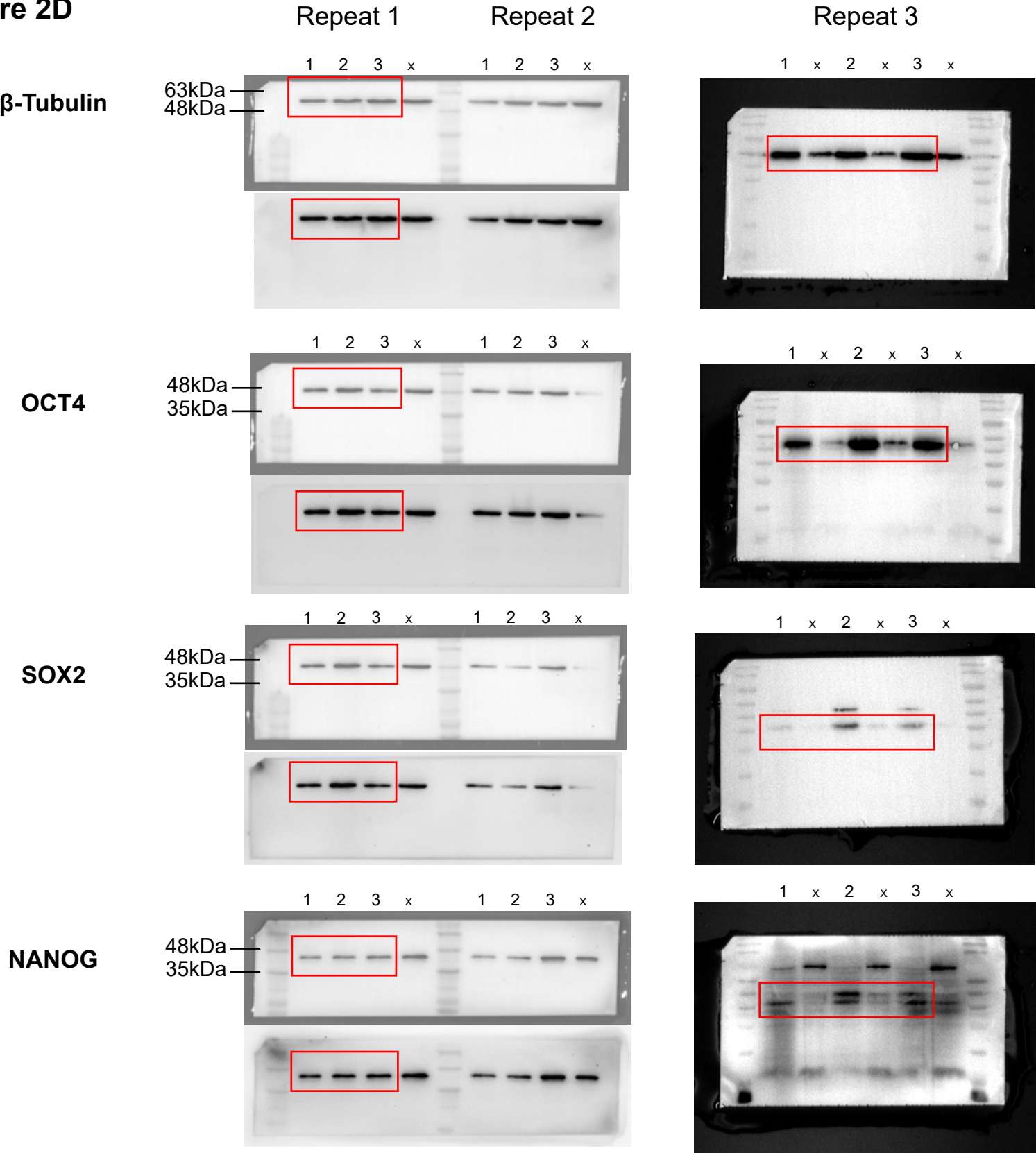

Figure 4D & Supplement Figure S1B

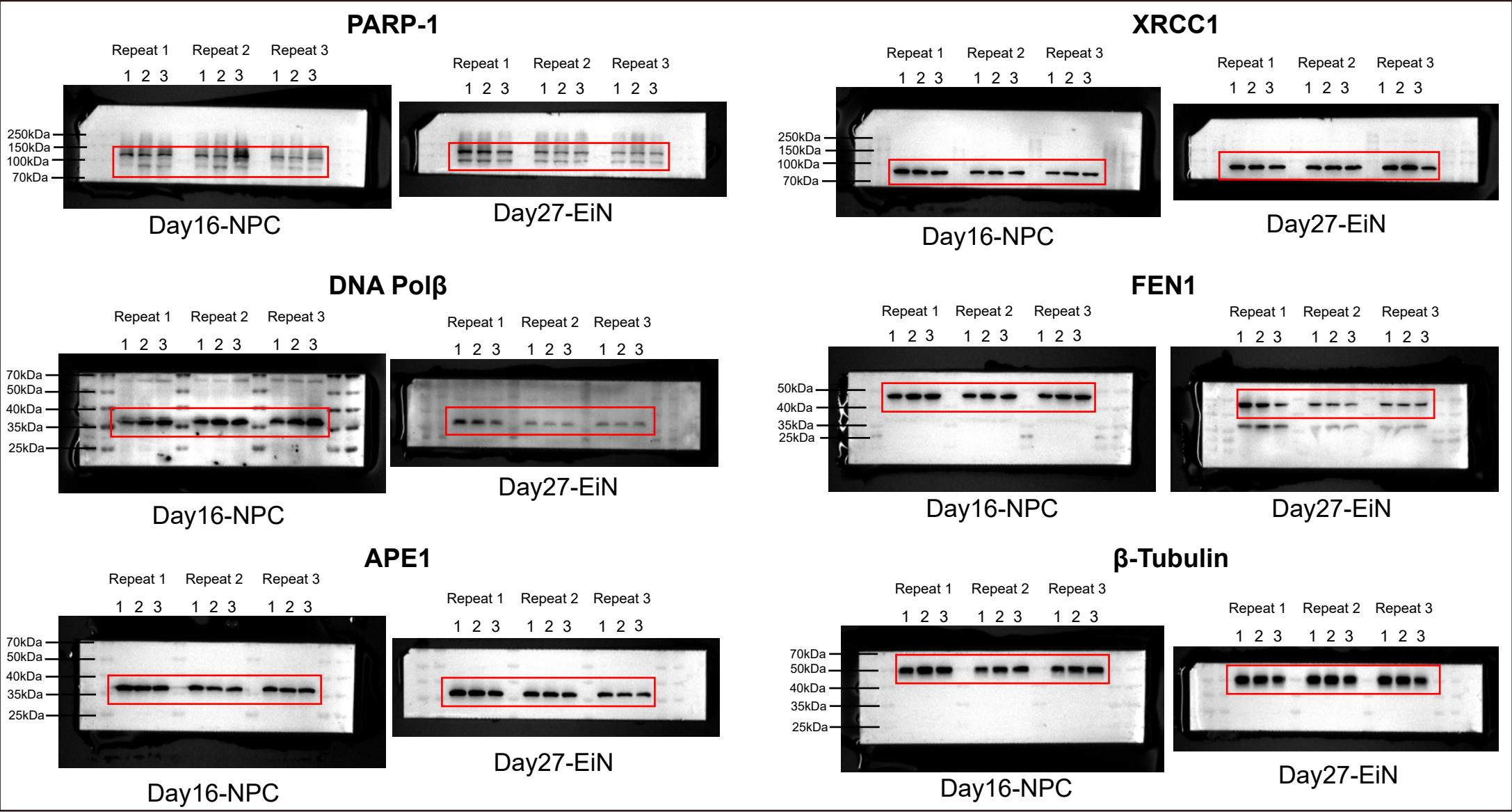

Supplement Figure S1A

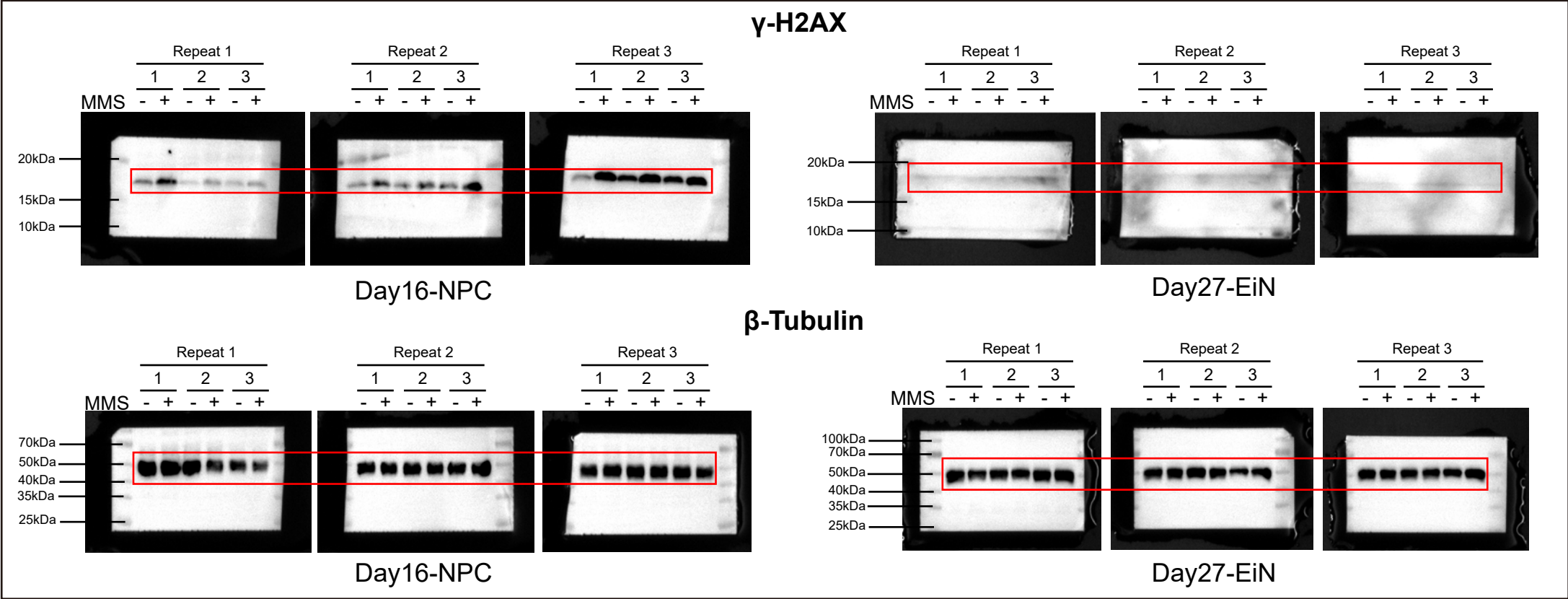

Legend: 1: Control 2: H201P 3: H201R

Supplement: Supplementary file 6 — Supplementary Uncropped Western Blot image [file 41420_2025_2723_MOESM6_ESM.pdf]
