# Supplementary material for: Induced pluripotent stem cells carrying novel APTX mutations presented defective neural differentiation with the accumulation of DNA single-strand breaks
Source: Cell Death Discov. 2025 Oct 24;11:481. doi: 10.1038/s41420-025-02723-2 (PMC12552585; doi:10.1038/s41420-025-02723-2)
Supplement: Supplementary file 3 — Supplementary Figure legends [file 41420_2025_2723_MOESM3_ESM.docx]

**Figure S1: DNA DSB and BER downstream proteins are not affected in iPSCs carrying *APTX* homozygous mutations during neural differentiation.**

1. Western blot analysis of γH2AX in NPCs on day 16 and in EiNs on day 27 after either 90 min incubation with MMS or not during neural differentiation. The experiments were repeated three times (n = 3, mean ± SD). Two-way ANOVA. There were no significant differences.
2. Western blot analysis of BER pathway proteins in NPCs on day 16 and in EiNs on day 27 during neural differentiation. The quantitative data of XRCC1, DNA Polβ, FEN1 and APE1 were analyzed. The experiments were repeated three times (n = 3, mean ± SD). One-way ANOVA. *, *P*<0.05; **, *P*<0.01.
